# Early-Onset Atrial Fibrillation Complicated by Substance Use Disorder and Psychiatric Comorbidities: A Case Report

**DOI:** 10.7759/cureus.67810

**Published:** 2024-08-26

**Authors:** Eleonora Achrak, Dominique Thurston, Mark Szkolnicki, Dragos Aconstantinesei, Larisa Mararenko

**Affiliations:** 1 Osteopathic Medicine, Touro College of Osteopathic Medicine, New York, USA; 2 Medicine, Brookdale University Hospital Medical Center, Brooklyn, USA

**Keywords:** general internal medicine, clinical anxiety, illicit drugs, atrial fibrillation (af), non valvular atrial fibrillation

## Abstract

Atrial fibrillation (AFib) is predominantly diagnosed in older adults, with early-onset AFib being relatively rare and often associated with preexisting heart conditions or due to hereditary familial AFib. This case report highlights the unusual diagnosis of AFib in an adolescent and the subsequent management complexities exacerbated by drug dependence and severe psychiatric comorbidities. The case shows the challenges in recognizing and treating AFib masked by psychiatric symptoms and substance abuse. A 33-year-old male with a history of AFib, diagnosed at 18, presented to the emergency department with symptoms suggestive of cardiac arrhythmia exacerbated by acute psychological stress and drug use. Despite a history devoid of classic cardiovascular risk factors, the patient developed drug dependence and psychiatric conditions over 15 years, potentially complicating and delaying effective AFib management. The patient’s treatment was further complicated by self-medication with illicit drugs, initially aimed at managing palpitations and anxiety, which may have masked or mimicked cardiac symptoms, thereby delaying appropriate AFib diagnosis and management. His recent hospitalization provided multidisciplinary interventions, including cardiology and psychiatric care, aiming to stabilize his cardiac and psychological conditions. This case illustrates the critical importance of considering early-onset AFib in differential diagnoses regardless of patient age and highlights the need for integrated care approaches in patients with complex comorbidities such as illicit drug use and severe mental health disorders. Moreover, it emphasizes the necessity of educating patients on the interactions between illicit drugs, psychiatric symptoms, and cardiac health. Early and accurate diagnosis, alongside comprehensive management, is crucial for improving outcomes in young patients with AFib complicated by noncardiac conditions. This report encourages further discussion and research into the management strategies for AFib in the context of psychiatric and substance abuse disorders.

## Introduction

Atrial fibrillation (AFib) is one of the most common cardiac arrhythmias, with a significant global burden impacting millions of lives each year. The Global Burden of Disease Study 2019 reported that AFib contributes notably to disability-adjusted life years, underscoring its widespread impact on public health [1]. While AFib is predominantly diagnosed in older adults, where its incidence increases markedly with age, the occurrence of AFib in younger individuals is relatively rare and is often associated with distinct risk factors and underlying conditions [2].

In younger patients, AFib can be particularly challenging to manage because it often arises in the context of specific risk factors that differ from those commonly seen in older populations. These can include genetic predispositions, such as familial AFib syndromes, congenital heart defects, or inherited ion channelopathies. Additionally, structural heart abnormalities, including atrial septal defects, hypertrophic cardiomyopathy, or mitral valve prolapse, are more frequently encountered in younger individuals presenting with AFib [2,3]. Noncardiac factors, such as hyperthyroidism, chronic inflammatory conditions, and significant lifestyle influences, including substance abuse, play a critical role in the pathogenesis of AFib in this demographic.

The pathophysiology of AFib in these cases involves a complex interplay of factors, including atrial remodeling, electrical conduction abnormalities, and systemic influences such as inflammation and oxidative stress [3,4]. In the context of substance use, particularly with stimulants like cocaine, there is an increased risk of AFib due to heightened sympathetic nervous system activity, which can precipitate arrhythmias by increasing heart rate and myocardial oxygen demand [5]. Psychiatric comorbidities, including anxiety and depression, further complicate this picture, as they are known to exacerbate the symptoms of AFib and complicate its management. The relationship between AFib and mental health issues is bidirectional, with each condition potentially worsening the other [6].

The novelty of this case lies in the intersection of early-onset AFib, substance use, and psychiatric disorders, which together create a complex clinical picture. Managing AFib in this context requires more than just addressing the arrhythmia; it necessitates a holistic approach that considers the psychological and behavioral factors at play. This report contributes to the growing body of literature on the diverse etiologies of AFib and highlights the importance of considering non-traditional risk factors in younger patients presenting with this condition.

## Case presentation

A 33-year-old Hispanic male with a known history of paroxysmal AFib, first diagnosed at age 18, presented to the emergency department in April 2024, complaining of nausea, vomiting, and palpitations. The patient had a complex medical history, including a 15-year history of substance use disorder and persistent anxiety. He reported poor management of his AFib and currently resides in a shelter. Despite being diagnosed with anxiety, he had not consistently treated his mental health condition, leading to ongoing psychological stress that likely exacerbated his cardiac symptoms. He disclosed that, although he had ceased crack cocaine use in January 2024, he continued to use marijuana and fentanyl.

**Figure 1:**
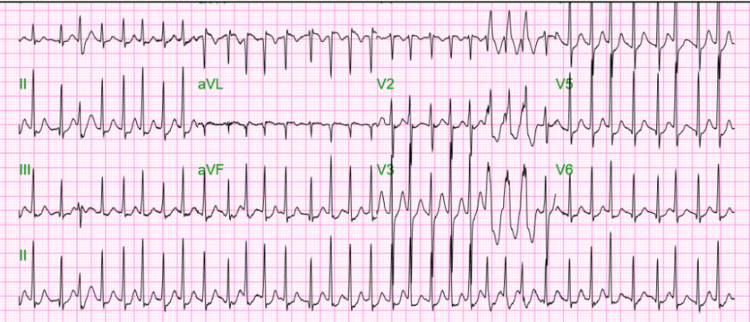
EKG at the emergency department visit showing AFib with RVR AFib, atrial fibrillation; RVR, rapid ventricular response

The day before this visit, the patient had seen his cardiologist due to worsening episodes of light-headedness and palpitations, which had intensified that morning, prompting his visit to the emergency department. On presentation, he was alert and oriented, with vital signs revealing a blood pressure of 97/65 mmHg, a pulse of 89 beats per minute, a respiratory rate of 20 breaths per minute, a temperature of 36.9 °C, and an oxygen saturation of 99% on room air. His physical examination was unremarkable, except for an irregularly irregular heartbeat. An initial EKG confirmed AFib with a rapid ventricular response (RVR), with a heart rate reaching 200 beats per minute (Figure 1).

Laboratory tests revealed sodium at 139 mmol/L, potassium at 3.4 mmol/L, and magnesium at 1.8 mg/dL, indicating mild hypokalemia and hypomagnesemia (Table 1). These electrolyte imbalances are often observed in patients with substance use disorders and can exacerbate cardiac arrhythmias like AFib. Immediate management in the emergency department included intravenous diltiazem, oral diltiazem at 30 mg, intravenous digoxin at 250 micrograms, and a magnesium sulfate injection at 1 g. These interventions led to an improvement in his heart rate. The patient was subsequently admitted to telemetry for symptomatic AFib with RVR.

**Table 1:** Initial blood test results in the emergency department

Test	Result	Reference range
Complete blood count		
WBC count	19.2	4.0-11.0 × 10^3^/µL
RBC count	5.05	4.20-5.70 × 10^6^/µL
Hemoglobin	15.3	13.5-17.5 g/dL
Hematocrit	43.50%	38.8-50.0%
Mean cell volume	86.1	80-96 fL
Mean cell hemoglobin	30.4	27-33 pg
Mean cell hemoglobin concentration	35.3	32-36 g/dL
Red cell distribution width	13.20%	11.5-14.5%
Platelet count	243	150-400 × 10^3^/µL
Mean platelet volume	10.8	7.4-10.4 fL
Basic metabolic panel		
Sodium	139	136-145 mmol/L
Potassium	3.4	3.5-5.1 mmol/L
Chloride	104	98-107 mmol/L
Carbon dioxide	20	22-29 mmol/L
Anion gap	15	3-11 mmol/L
Blood urea nitrogen	15	8-21 mg/dL
Creatinine	1.2	0.7-1.3 mg/dL
Glucose	158	70-99 mg/dL
Magnesium	1.8	1.9-2.7 mg/dL
Lactate	2.3	0.5-2.2 mmol/L
Liver panel		
Estimated glomerular filtration rate	81.9	>90 mL/min/1.73m^2^
Phosphorus	2	2.5-4.5 mg/dL
Cardiac profile		
Troponin	<5.0	<5.0-19.7 pg/ml

His hospital course included further assessments, beginning with a transthoracic echocardiogram (TTE) conducted one day post-admission, which revealed a normal left ventricular ejection fraction of 55% and a normal left ventricular size of 2.83 cm. Cardiac enzymes remained within normal limits (<5 pg/ml), indicating the absence of acute coronary ischemia. The patient’s medication was adjusted to oral diltiazem 240 mg, effectively stabilizing his heart rate and palpitations.

**Figure 2:**
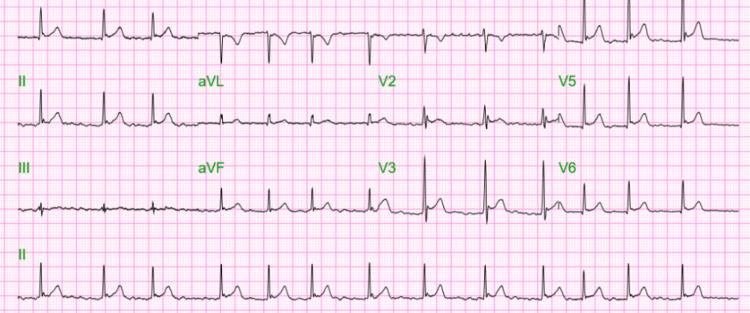
EKG displaying AFib AFib, atrial fibrillation

Given the recurrent nature of his AFib and his young age, an electrophysiology (EP) evaluation was performed to explore the underlying etiology of his condition. During the consultation, he underwent additional cardiac diagnostic tests, including a repeat 12-lead EKG (Figure 2) and continuous telemetry monitoring. With a rate-control approach, his AFib spontaneously terminated within a few days, although additional intervention with calcium channel blockers was necessary to maintain sinus rhythm. Notably, ST elevations were observed in leads II and V2-V6, consistent with early repolarization and a prominent elevated J point.

Other potential cardiopathies and infections were ruled out through comprehensive cardiac biomarker analysis, blood work to exclude thyroid abnormalities, and repeated echocardiograms and EKGs.

Twenty-four-hour telemetry monitoring captured several episodes of paroxysmal AFib, correlating with the patient’s reported symptoms of palpitations and anxiety. Although no significant ventricular arrhythmias were observed, the frequent AFib episodes highlighted the need for more aggressive intervention. The consulting electrophysiologist recommended an arrhythmic risk assessment and discussed the feasibility of an AFib ablation procedure on an outpatient basis, considering the patient’s young age and the recurrent nature of his arrhythmia. Genetic testing was also suggested to explore the possibility of an underlying familial arrhythmia syndrome, given the early onset of his AFib. A follow-up EP appointment was scheduled for two weeks post-discharge to review the genetic testing results and finalize the decision regarding ablation. The patient was advised to maintain a log of his symptoms and to continue taking diltiazem as prescribed. Coordination between the EP team and the patient’s cardiologist was established to ensure a streamlined approach to his care, with a focus on a tailored therapy plan that addressed both his AFib and broader health concerns. The patient was discharged three days after admission.

Two days after discharge, the patient experienced another episode of AFib with RVR and an elevated heart rate of 170 beats per minute. He presented to the emergency department, where he was managed with a 5 mg intravenous push of metoprolol. Despite remaining hemodynamically stable throughout the episode, he became agitated and anxious, which was managed with 2 mg of oral lorazepam. Plans were made for discharge the following day, with outpatient follow-up scheduled with cardiology and addiction services. His discharge medications included diltiazem 240 mg daily, and arrangements were made for ongoing substance abuse treatment. The patient reported feeling comfortable on diltiazem and declined additional medication for his anxiety.

**Figure 3:**
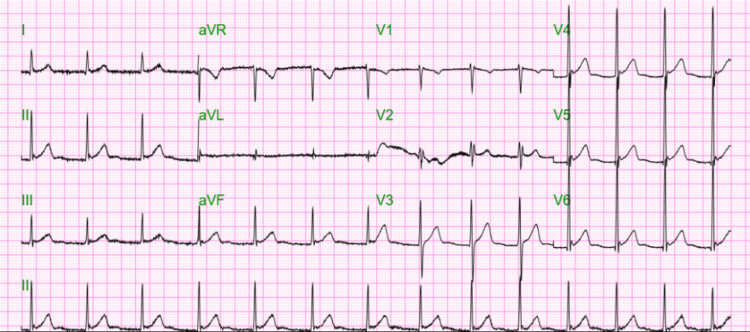
EKG showing an accelerated junctional rhythm during readmission

However, one week post-discharge, the patient was readmitted to the psychiatric unit after presenting with severe anxiety, abdominal pain, and suicidal ideation. He expressed overwhelming stress related to his medical condition and revealed a plan to overdose on diltiazem and fentanyl. During this admission, he was treated with intravenous and intramuscular formulations of haloperidol, midazolam, and metoclopramide, along with a 1L bolus of Ringer’s lactate. His laboratory workup showed an elevated white blood cell count of 12.9 µL, likely secondary to stress, and negative troponin levels (Table 2). The EKG during this episode showed an accelerated junctional rhythm, with no evidence of AFib (Figure 3).

**Table 2:** Blood test results from the psychiatric unit

Test	Result	Reference range
Complete blood count		
WBC count	12.9	4.0-11.0 × 10^3^/µL
RBC count	4.96	4.20-5.70 × 10^6^/µL
Hemoglobin	15.3	13.5-17.5 g/dL
Hematocrit	42%	38.8-50.0%
Mean cell volume	84.6	80-96 fL
Mean cell hemoglobin	30.9	27-33 pg
Mean cell hemoglobin concentration	36.5	32-36 g/dL
Red cell distribution width	12.90%	11.5-14.5%
Platelet count	221	150-400 × 10^3^/µL
Mean platelet volume	10.6	7.4-10.4 fL
Basic metabolic panel		
Sodium	138	136-145 mmol/L
Potassium	3.7	3.5-5.1 mmol/L
Chloride	103	98-107 mmol/L
Carbon dioxide	19	22-29 mmol/L
Anion gap	16	3-11 mmol/L
Blood urea nitrogen	17	8-21 mg/dL
Creatinine	1.2	0.7-1.3 mg/dL
Glucose	106	70-99 mg/dL
Magnesium	1.8	1.9-2.7 mg/dL
Lactate	1.7	0.5-2.2 mmol/L
Liver panel		
Estimated glomerular filtration rate	81.9	>90 mL/min/1.73m^2^
Phosphorus	2.3	2.5-4.5 mg/dL
Cardiac profile		
Troponin	<5	<5.0-19.7 pg/ml

The patient became an imminent danger to himself and was therefore admitted to the psychiatric unit.

## Discussion

This case report examines the clinical presentation of early-onset AFib in a patient with a long history of substance use and untreated psychiatric comorbidities. The patient’s 15-year history of substance abuse, coupled with persistent anxiety that has been inconsistently managed and largely untreated, significantly complicated his overall health and the management of his AFib. Importantly, the patient, a Spanish speaker, experienced significant communication difficulties, even when interacting with Spanish-speaking healthcare providers. This added layer of complexity likely contributed to his inconsistent care and the recurrence of his AFib episodes.

The recurrence of AFib episodes despite ongoing medical therapy for the arrhythmia indicated a need for a more tailored approach to intervention. Pulmonary vein isolation (PVI) is often considered in such cases, particularly in younger patients who continue to experience symptomatic AFib despite optimal pharmacological management. PVI, typically performed via catheter ablation, targets areas of the heart responsible for initiating erratic electrical signals and has been shown to be an effective strategy for reducing AFib recurrence [7]. However, in cases where AFib is secondary to underlying conditions like substance abuse and psychiatric disorders, catheter ablation is not proactively recommended as a first-line treatment. In this patient’s case, with AFib being driven by substance use and mental health issues, the focus was placed on managing these underlying conditions rather than pursuing ablation. Consequently, the patient did not undergo the procedure.

However, in reviewing this case, it is evident that the patient’s overall treatment approach may not have been sufficiently aggressive or comprehensive. The frequent recurrence of AFib, along with the deterioration of his mental status over a few weeks, suggests that his care may have lacked the necessary intensity and coordination. This case highlights the critical need for a more integrated care model, where both psychiatric and cardiovascular issues are managed simultaneously, with careful consideration of how each impacts the other.

The role of psychological factors in AFib is increasingly recognized, with studies showing that anxiety and depressive symptoms can exacerbate AFib, particularly in underprivileged populations [8]. The impact of psychosocial stressors on AFib progression is well-documented, with prospective studies indicating that such stressors can negatively influence AFib management and outcomes, particularly among women. Despite the evidence linking psychological distress to AFib, there is a paucity of evidence-based interventions targeting the behavioral aspects of AFib management. Non-pharmacological approaches, such as cognitive behavioral therapy, biofeedback, and relaxation techniques, have shown promise in modulating the autonomic nervous system and improving symptom management in AFib patients [9,10]. However, these interventions are not yet widely implemented in clinical practice, particularly in patients like this one, who are dealing with a complex interplay of psychiatric and cardiac issues.

In this case, the patient’s anxiety has remained largely untreated, which likely contributed to the exacerbation of his AFib symptoms. The absence of anxiety management is concerning, as untreated anxiety can lead to increased sympathetic nervous system activity, which is a known trigger for arrhythmias. Additionally, the combination of untreated anxiety and substance use, particularly ongoing fentanyl use, further complicates the clinical picture. The risk of respiratory depression, sedation, and the masking of cardiac symptoms due to drug use adds another layer of complexity to his care.

The diagnostic workup during the patient’s hospitalization included TTE and telemetry monitoring, which were instrumental in guiding subsequent treatment decisions. The TTE showed normal ventricular function, suggesting that despite the recurrent nature of his AFib, the patient had not yet developed significant ventricular impairment or heart failure, which would have further complicated his management. The absence of significant valvular disease further simplified the approach, allowing the focus to remain on managing the arrhythmia rather than addressing structural heart issues.

Given the complexity of this case, where AFib, anxiety, substance use disorder, and language barriers intersect, a multidisciplinary approach to management is essential. This involves not only direct rhythm control strategies and anticoagulation but also careful psychiatric management and behavioral interventions aimed at reducing the triggers of both anxiety and AFib. A coordinated care model, possibly involving a multidisciplinary team of cardiologists, psychiatrists, addiction specialists, and language services, could improve outcomes for such patients.

## Conclusions

This case report highlights the complexities of managing early-onset AFib, complicated by substance use and psychiatric conditions. Diagnosed at 18, the patient’s treatment was challenged by recurrent emergency visits and noncardiac comorbidities. His case shows the importance of a holistic approach that addresses cardiac symptoms and broader psychosocial factors.

Integrating cardiology and psychiatric care is essential for managing such complex cases. This patient’s story emphasizes the need for patient education on the interactions between illicit drugs, mental health, and cardiac health. Enhanced understanding can improve treatment adherence and reduce emergency visits. This report advocates for ongoing research and innovative management strategies for AFib, especially in patients with atypical histories. These include multidisciplinary management of such patients from cardiology and psychiatry simultaneously versus clearing the patient separately, as well as research focused on adolescent drug-induced cardiac arrhythmias and early onset atypical cardiac arrhythmias. Comprehensive care approaches are crucial for improving both immediate health outcomes and long-term quality of life for these vulnerable populations.
